# Postthoracotomy Ipsilateral Shoulder Pain: A Literature Review on Characteristics and Treatment

**DOI:** 10.1155/2016/3652726

**Published:** 2016-11-28

**Authors:** Fardin Yousefshahi, Oana Predescu, Melissa Colizza, Juan Francisco Asenjo

**Affiliations:** ^1^Department of Anesthesia, Tehran University of Medical Sciences, Tehran, Iran; ^2^Montreal General Hospital, McGill University Health Centre, Montreal, QC, Canada; ^3^Department of Anesthesia, Montreal General Hospital, McGill University Health Centre, Montreal, QC, Canada; ^4^Department of Anesthesia, McGill University Health Centre, Montreal, QC, Canada

## Abstract

*Context.* Postthoracotomy Ipsilateral Shoulder Pain (IPS) is a common and sometimes intractable pain syndrome. IPS is different from chest wall pain in type, origin, and treatments. Various treatments are suggested or applied for it but none of them is regarded as popular accepted effective one.* Objectives.* To review data and collect all present experiences about postthoracotomy IPS and its management and suggest future research directions.* Methods.* Search in PubMed database and additional search for specific topics and review them to retrieve relevant articles as data source in a narrative review article.* Results.* Even in the presence of effective epidural analgesia, ISP is a common cause of severe postthoracotomy pain. The phrenic nerve has an important role in the physiopathology of postthoracotomy ISP. Different treatments have been applied or suggested. Controlling the afferent nociceptive signals conveyed by the phrenic nerve at various levels—from peripheral branches on the diaphragm to its entrance in the cervical spine—could be of therapeutic value. Despite potential concerns about safety, intrapleural or phrenic nerve blocks are tolerated well, at least in a selected group of patient.* Conclusion.* Further researches could be directed on selective sensory block and motor function preservation of the phrenic nerve. However, the safety and efficacy of temporary loss of phrenic nerve function and intrapleural local anesthetics should be assessed.

## 1. Introduction

Open thoracotomy surgeries constitute very painful procedures [1, 2]. While thoracic epidural analgesia may help control the incisional component of the pain, an excruciating postthoracotomy Ipsilateral Shoulder Pain (ISP) could undermine pain management in the postthoracotomy patient [3]. Shoulder pain is also common in abdominal surgery with some similarities, but its management seems somewhat different. Very little specific data exists about ISP in the literature [4]. Furthermore, the majority of studies about ISP are not well designed clinical trials. The present literature review is an attempt to organize the present clinical knowledge and comprehension about postthoracotomy ISP and its management, to orient future research directions.

## 2. Material and Method

We performed a search in PubMed database for “thoracotomy”, “pain”, and “shoulder” and applied filters to obtain human studies only. All relevant studies related to postthoracotomy shoulder pain, released until November 2015, were reviewed in detail. Further searches were performed based on our primary findings in related topics. The retrieved articles were organized in order to prepare a narrative review article focused on postthoracotomy ISP treatment.

## 3. Frequency and Importance

In the presence of good thoracic epidural analgesia, ISP may contribute to all the pain experienced by thoracotomy patients. As, by blocking the phrenic nerve and in presence of a competent epidural analgesia, most patients do not report as much pain [5].

ISP could affect 31% to 85% of patients after thoracic surgery [5–13]. Even, one study reported a prevalence of up to 97% [9]. ISP could impair effective breathing and the effectiveness of physical therapy in the early postoperative period [14].

## 4. Pain Characteristics

Postthoracotomy ISP is limited to the operative side [11]. Postthoracic surgery ISP is reported as moderate to severe in the majority of patients [3, 6, 8, 15–18]. In approximately half of the patients, ISP was located at the posterior side of the shoulder [6], but it may be felt around the deltoid region [16, 18], the superior-posterior area of the shoulder [3], or the lateral third of the clavicle [17]. All those areas share innervation from C4 and C5 with the diaphragm.

ISP onset could be as early as the first postoperative hour [9, 11]. With treatment, the pain severity will decrease over the first 4 to 6 hours [5, 11, 13].

Less intense pain could extend to the second postoperative day [7, 15].

ISP is often described as dull, aching pain [3, 16, 18]. While the majority of reports define the pain as independent from movement [3, 11, 16, 18], other reports describe ISP to be aggravated by movement [17]. However, effective treatment of pain will decrease ISP severity, both during rest and cough [9].

## 5. Etiology

As ISP may be aggravated by movement, it is suggestive for a somatic rather than neuropathic origin of pain [6]. However, both inflammatory [3] and neuropathic [19] behavior had been suggested for ISP.

There are many unproved or partially proved hypotheses regarding the etiology of ISP including transection of major bronchi, ligament distraction or strain as the result of surgical retraction, shoulder joint strain from intraoperative mal-positioning, myofascial involvement, irritation of the pleura by chest drains, and referred pain from irritation of the pericardium, mediastinum, or diaphragmatic surface [3, 5, 11, 17, 20, 21].

Transection of the major bronchi was suggested in a study as the cause of ISP after thoracic surgeries in 1993 [3]. However, it was not confirmed in other studies [6].

Severe incisional pain of thoracotomy could be the result of surgical trauma to some major elements in the chest wall, including the latissimus dorsi muscles and ribs. It had been suggested that the shoulder pain after thoracotomy could be caused by strain of ligaments and muscles of scapula [17], arcuation of patient in lateral decubitus position [13], or ligament injury following rib retraction [20]. Furthermore, the localization of ISP to the posterior side of shoulder, in a majority of patients, could suggest the muscle strain around the scapula as the cause of ISP [6].

The relationship between a longer duration of surgery in the lateral decubitus position and ISP has been suggested as an explanation for the role of strain of the shoulder joint capsule and ligaments in the etiology of ISP [6, 20]. Patients with a higher Body Mass Index (BMI) report an increased prevalence of postthoracotomy IPS, representing a possibly higher strain on the shoulder and thus supporting this hypothesis in another study [22]. Meanwhile, the distraction of the ipsilateral shoulder joint was suggested as a possible cause for the limited effect of an intrapleural block, the supraclavicular nerve block was ineffective in palliating ISP [13], which suggests it is not a common cause of ISP.

Myofascial involvement has also been reported to contribute at least partially to postthoracotomy shoulder pain [21].

Another hypothesis is that ISP is the result of referred pain from the irritated pericardium, mediastinum, or pleural surface of the diaphragm, transmitted via the phrenic nerve [5, 11, 23]. Many studies support this hypothesis, since the intraoperative infiltration of the phrenic nerve at the level of the diaphragm could diminish the incidence of ISP significantly [5, 9, 10]. Furthermore, there is a reduction in the incidence of ISP, following the block of phrenic nerve as a side effect of stellate ganglion block [16] and interscalene brachial plexus block [7, 17, 18, 24]. There is also a report on effective treatment of postthoracotomy ISP by direct blocking the phrenic nerve at the neck [17].

The phrenic nerve is a mixed nerve that contains afferent supply from the subdiaphragmatic peritoneum, liver, spleen, diaphragm, lower part of the pleura, and pericardium [23, 25]. It is originating from 3rd, 4th, and 5th cervical nerve roots. Stimulation of these afferent nerves could translate into referred pain, which is perceived clinically as neck and shoulder pain. The diaphragmatic afferents comprise small unmyelinated C-fibers, which have a high excitation threshold [26]. This mechanism is supported by the occurrence of IPS in the presence of phrenic nerve stimulation devices [5, 27].

Pleural irritation by chest drains is considered to be the most probable cause of ISP in postthoracotomy patients. A study by Pennefather et al. suggested that, in some patients, ISP could be the result of direct irritation of the apical or basal pleura by chest tubes or by blood [11]. However, intrapleural administration of bupivacaine via basal chest tubes could not relieve the shoulder pain [11].

## 6. Risk Factors

Predisposing and risk factors for developing ISP are not well known. However, in a prospective observational study by Bunchungmongkol et al., the potential risk factors for developing ISP were surgery performed by a thoracotomy approach compared with Video-Assisted Thoracoscopic Surgeries (VATS) (risk ratio: 2.12, 95% confidence interval: 1.16–3.86, *p* = 0.014) and surgery duration >2 hours (risk ratio: 1.61, 95% confidence interval: 1.07–2.44, *p* = 0.023) [6]. In a retrospective study by Misiołek et al., higher Body Mass Index (BMI), thoracotomy versus VATS approaches, and epidural catheter level lower than T5 were found to be associated with a higher prevalence of IPS [22]. In this study 4.35% of patients with an epidural catheter placed at T5 or higher developed ISP while 40.47% of patients with epidural catheter placed lower than T5 developed it [22]. However, coincidence of open thoracotomy, long duration of surgery, and high BMI could not be ignored.

The direct relationship between long duration of surgery and ISP occurrence [20] and also superiority of VATS on thoracotomy [28] were reported by other authors, too.

## 7. Treatments

ISP is often difficult to manage, as it is relatively resistant to parenteral opioids and increases in epidural infusion rates [5, 15].

Some reports address the effectiveness of Nonsteroidal Anti-Inflammatory Drugs (NSAIDs), such as ketorolac and indomethacin for postthoracotomy ISP treatment [3, 6–8]. Though others report only a partial efficacy of NSAIDs [5]. However, the efficacy of NSAIDs still requires further investigation as well as consideration to contraindications to the use in several populations.

Preemptive, regular prescription of acetaminophen during the first 48 hours from thoracotomy may decrease ISP [15]. While gabapentin is effective in prevention of shoulder pain after abdominal surgeries [29], it was not effective in treatment of postthoracotomy IPS, even as preemptive analgesia [19, 31]. Other popular molecules used for neuropathic pain (i.e., pregabalin) are regarded to be effective for treatment of ISP by other authors [27, 30].

Systemic ketamine is reported to be effective to decrease the acute postthoracotomy pain [32–36]. Epidural ketamine is also reported to be effective in prevention of acute postthoracotomy pain and development of chronic postthoracotomy pain syndrome [37]. However, other studies could not find such a beneficial effect on chronic pain for systemic [32, 35, 36] or epidural ketamine [36]. However, none of these studies address ISP as an outcome in their studies.

Thoracic epidural analgesia likely provides optimal analgesia for thoracotomy incision, along with a reduction in morbidity and mortality after open thoracic surgeries [38–41]. However, a perfectly adequate thoracic epidural analgesia for the incisional site is not effective in preventing postthoracotomy ISP [6, 8, 16]. ISP is often resistant to increases in the epidural infusion rate [5, 15]. However, in a recent study, epidural catheters located in spaces higher than T5 were effective in preventing postthoracotomy ISP [22].

Intraoperative local anesthetic infiltration of the phrenic nerve at the level of the diaphragm could significantly reduce the incidence of ISP [5, 7, 9, 10]. However, the benefit of this technique is limited by its short duration of effect and impossibility to be repeated [5]. Using long acting local anesthetics to block the phrenic nerve may provide longer analgesia, but there are concerns about longer hemidiaphragmatic weakness and its consequences in patients that require full function of the diaphragm after surgery [11]. But in another study, intraoperative phrenic nerve infiltration with 10 mL of ropivacaine 0.2% was shown to be effective and without additional compromise of oxygenation [9].

Stellate ganglion block [7, 16] and interscalene brachial plexus block [7, 17, 18, 24] are effective in reducing postthoracotomy ISP, possibly because the phrenic nerve is often blocked concomitant on the same side. Interscalene blockade in treatment of ISP has yielded better results than diclofenac; however the phrenic nerve motor function is also blocked most times [7]. There is also a report of effective treatment of postthoracotomy ISP by direct blockade of the phrenic nerve, after its inadvertent identification during attempt to locate brachial plexus [17]. The lower prevalence of postthoracotomy ISP when an epidural catheter is inserted higher than T5 is considered as supportive of the role of phrenic nerve in treatment of ISP [22].

ISP after VATS is reported in few studies [4, 6, 28]. Considering the smaller surgical incisions and the shorter duration of the procedures, VATS is helpful in reducing early postoperative incisional pain [6, 28].

The intrapleural block, that is, local anesthetic injection between visceral and parietal pleura, is effective in relieving the shoulder pain after cholecystectomy [42]. While there are reports which address safety and advantages of intrapleural block in postthoracotomy patients [43–50], most other studies showed little or no benefit for ISP [11, 51–55]. As an interesting finding, intrapleural block could not decrease epidural analgesia requirement and have little effect on intercostal incisional pain after thoracotomy, too [11, 57]. The difference between the results with cholecystectomy versus thoracotomy could be explained by the presence of blood and air in the pleural space after thoracotomy, which limits effective local anesthetic spread [11]. Local anesthetic loss via the chest tubes, insufficient volume of local anesthetic, and dilution of local anesthetic by blood or pleural effusion fluid and presence of an accessory phrenic nerve have been considered as possible causes of unsuccessfulness of intrapleural block [11, 45, 54]. Moreover, a phrenic nerve block at the diaphragm level may or may not be necessarily effective at controlling ISP in all patients [5]. Additionally, it has been shown that sensory and sympathetic block after intrapleural analgesia is incomplete, even in abdominal surgeries [58].

As mediastinal pleura and pericardium receive some sensory innervation from the phrenic nerve [23], it is possible that a more proximal block is necessary to block these branches [11].

Interestingly, intrapleural morphine was shown to be more effective in pain control when compared to intravenous morphine in postthoracotomy patients. Moreover, plasma levels of morphine were lower and respiratory rate was higher in intrapleural route [59]. There is no report about ISP in this study.

Intrapleural block was used safely and successfully for pain management after VATS and thoracotomy, either as intermittent bolus doses or as continuous infusion [60–63]. It has been used in long-term pain management of cancer patients, chronic thoracotomy pain with intermittent injection [64–67], chronic abdominal or pancreatic pain [68–70], chest wall trauma, rib fractures [71], and even postoperative coronary artery bypass graft (CABG) [72–74]. However, none of these applications regards ISP after thoracotomy or VATS.

While diaphragmatic (phrenic nerve) block could be induced by intrapleural block [57, 75], required doses in the postthoracotomy setting are potentially large and within the toxic range. Furthermore, a large irritated pleural space after thoracotomy could enhance local anesthetic absorption [76, 77] and cause systemic toxicity. For example, while the toxicity risk increases with bupivacaine plasma levels above 2–4 *μ*g·mL^−1^, peak plasma concentrations of bupivacaine in postthoracotomy patients who received 1.5 mg·kg^−1^ for an intrapleural block (40 mL of bupivacaine 0.25% in a 70 kg patient) have been shown to be 1.50 *μ*g·mL^−1^ [44]. However, adding epinephrine may potentially lower the plasma concentration of local anesthetics and enhance duration of effect [43, 78]. In one report, preemptive preoperative intrapleural block results in a blunted hemodynamic response to surgery and decrease of anesthetic requirements, with significantly lower arterial blood pressure and heart rate, likely secondary to the sympathetic blockade versus concomitant isoflurane use [79]. Other side effects of intrapleural block include respiratory arrest, which was reported in a narcotized patient [80] and Horner's syndrome [81]. Nevertheless, intrapleural analgesia resulted in the improvement of respiratory performance as well as a decrease in pulmonary complications in postoperative CABG patients with concomitant chronic obstructive pulmonary disease (COPD) [74], as previously reported in postthoracotomy patients [44, 50, 82].

Repositioning of the chest tubes should be considered when direct irritation of the apical pleura is suspected as a cause for ISP based on chest X-ray assay [11].

In a 2002 study, suprascapular nerve block was not successful in controlling postthoracotomy ISP [13], but other studies did report a beneficial effect in patients with postthoracotomy ISP [56], at least in the subgroup of patient with localized musculoskeletal tenderness in shoulder [12, 17]. However its effect in postthoracotomy ISP seems to be of less magnitude compared to the phrenic nerve block [10].

Both intercostal nerve block [83] and paravertebral block [84–89] are considered to decrease the postthoracotomy pain. However, they were not effective in prevention of ISP [83] or their efficacy on ISP is not reported.

ISP could lead to shoulder dysfunction after thoracotomy and postoperative physiotherapy could improve shoulder function [90], provided that the pain is first under control.

Acupuncture in conjunction with other treatments is reported to be helpful for postabdominal laparoscopy shoulder pain [91], but there is no data about such complementary treatments for postthoracotomy ISP.

Table 1 is summarizing advantages and disadvantages of different techniques of management of ISP.

## 8. Future Directions

The paucity of well-organized studies on postthoracotomy ISP, along with the high prevalence, severity of pain, and its suboptimal response to common treatments mandates further well designed trial studies that compare potentially effective and safe treatments. Different sources for pain after thoracotomy require the application of different modalities of pain management, simultaneously. These could be high thoracic epidural anesthesia (at T5 or above) and another modality or level of epidural to cover ISP. Current available data are in favor of safety and efficacy of intrapleural analgesia and phrenic nerve blocks. They could be subjects of new researches.

Phrenic afferent stimulation could be perceived as referred pain in the neck and shoulder. This mechanism is supported by ISP sensation in patients with phrenic nerve stimulation devices [27]. This afferent supplies consist in small unmyelinated C-fibers, which have a high excitation threshold [26]. Accordingly, neuropathic pain medications such as pregabalin, gabapentin, and duloxetine are reported to be useful for ISP treatment [27]. Therefore, future research should involve finding practical ways to perform differential sensory block of phrenic nerve at neck via specific TENS devices or by C-fiber targeted medications for treatment of ISP.

## Figures and Tables

**Table 1:** Advantages and disadvantages of different techniques of management of ISP.

Technique	Efficacy in Ipsilateral Shoulder Pain (ISP)	Advantages	Disadvantages	Comments
Nonsteroidal Anti-Inflammatory Drugs *(NSAIDs)*	Yes [3, 6–8] Partial effect [5]	Simplicity of use Cross-effectivity for other pain syndromes	Unwanted side effects and contraindications Limited level of efficacy	Considered as coanalgesia

*Acetaminophen*	Yes [15]	Simplicity of use Cross-effectivity for other pain syndromes May be effective as preemptive analgesia Well tolerated	Limited level of efficacy	Considered as coanalgesia

*Gabapentin*	Yes [27, 29, 30] No [19, 31]	Simplicity of use Cross-effectivity for other pain syndromes	More effective in ISP in abdominal surgeries May cause dizziness or sedation	Considered as coanalgesia

*Systemic ketamine *	There is no report(s) about ISP Several reports about efficacy thoracotomy pain management [32–37] or against it [32, 35, 36]	Simplicity of use Cross-effectivity for other pain syndromes May be effective in prevention of chronic post thoracotomy pain development	May cause dizziness, sedation, or other neurologic or cardiac side effects	More researches are required

*Thoracic epidural*	Optimal analgesia for incisional pain [38–41] At levels higher than T5 effective on ISP [22] Not effective for ISP [5, 6, 8, 15, 16]	Excellent pain relief for incisional pain	Limited or no efficacy on ISP Hemodynamic effects Invasive method Needing further assessments	Recommended for all thoracotomy patients More research about the efficacy of epidurals higher than T5 in management of ISP

*Intraoperative local anesthetic infiltration of the phrenic nerve at the level of the diaphragm *	Yes [5, 7, 9, 10]	Significant effect on prevention of ISP Simple and effective There are reports about its safety	Short duration of effect Impossibility to repeat Concerns about hemidiaphragmatic weakness Needing surgical access	Recommended as a preventive effective method in otherwise healthy patients Need more assessments of safety at least in special subgroups of patient Needs development of technique and medication selection

*Stellate ganglion block *	Yes [7, 16]	Possible to repeat	Invasive Unwanted effects and side effects of stellate ganglion block	To be considered as a possibility to treatment of ISP in special patient Needing development of technique and medication selection

*Interscalene brachial plexus block*	Yes [7, 17, 18, 24]	Possible to repeat	Invasive Unwanted effects and side effects of brachial plexus block	To be considered as a possibility to treatment of ISP in special patient including multiple trauma of shoulder or arm Needing development of technique and medication selection

*Direct blockade of the phrenic nerve*	Yes (inadvertent) [17]	Possible to repeat	Invasive Concerning about hemidiaphragmatic weakness	Needing more assessments of safety and efficacy Needing development of technique and medication selection

*Intrapleural block*	Yes [42–50] No [11, 51–55]	Safe and easy Could be repeated as needed Could be perform via chest tubes	Some systemic side effects of intrapleural medications No effect on incisional pain or epidural analgesia requirement Sometimes incomplete block	Could be considered as the first option in early postoperative phase, when clots, secretions, and adhesion bonds are minimal and ISP have the highest severity Needing more studies for development of technique and medication selection

Suprascapular nerve block	Yes [10, 12, 17, 56] No [13]	Possible to repeat	Invasive Unwanted effects and side effects of Less effectiveness in compare with phrenic nerve block	Needing more assessments of safety and efficacy To be considered as a possibility to treatment of ISP in special patient including multiple trauma of shoulder or arm
